# Changes in the Sodium Content of Australian Processed Foods between 1980 and 2013 Using Analytical Data

**DOI:** 10.3390/nu9050501

**Published:** 2017-05-15

**Authors:** Felicity Zganiacz, Ron B. H. Wills, Soumi Paul Mukhopadhyay, Jayashree Arcot, Heather Greenfield

**Affiliations:** 1 Formerly an Honours student within the Food Science and Technology Group, School of Chemical Engineering, UNSW, Sydney, NSW 2052, Australia; felicity.zganiacz@live.com; 2 Department of Food Technology, Faculty of Science and Information Technology, University of Newcastle, Ourimbah, NSW 2258, Australia; ron.wills@newcastle.edu.au; 3 School of Agricultural and Wine Sciences, Charles Sturt University, Wagga Wagga, NSW 2678, Australia; Smukhopadhyay@csu.edu.au; 4 Food Science and Technology Group, School of Chemical Engineering, UNSW, Sydney, NSW 2052, Australia; h.greenfield@unsw.edu.au

**Keywords:** sodium, processed foods, cardiovascular disease, food reformulation, food label accuracy

## Abstract

The objective of this study was to obtain analytical data on the sodium content of a range of processed foods and compare the levels obtained with their label claims and with published data of the same or equivalent processed foods in the 1980s and 1990s to investigate the extent of any change in sodium content in relation to reformulation targets. The sodium contents of 130 Australian processed foods were obtained by inductively coupled plasma optical emission spectrometry (ICP-OES) analysis and compared with previously published data. The sodium content between 1980 and 2013 across all products and by each product category were compared. There was a significant overall sodium reduction of 23%, 181 mg/100 g (*p* <0.001, 95% CI (Confidence Interval), 90 to 272 mg/100 g), in Australian processed foods since 1980, with a 12% (83 mg/100 g) reduction over the last 18 years. The sodium content of convenience foods (*p <* 0.001, 95% CI, 94 to 291 mg/100 g) and snack foods (*p* = 0.017, 95% CI, 44 to 398 mg/100 g) had declined significantly since 1980. Meanwhile, the sodium contents of processed meats (*p =* 0.655, 95% CI, −121 to 190) and bread and other bakery products (*p* = 0.115, 95% CI, −22 to 192) had decreased, though not significantly. Conversely, the sodium content of cheese (*p* = 0.781, 95% CI, −484 to 369 mg/100 g) had increased but also not significantly. Of the 130 products analysed, 62% met Australian reformulation targets. Sodium contents of the processed foods and the overall changes in comparison with previous data indicate a decrease over the 33 years period and suggest that the Australian recommended reformulation targets have been effective. Further sodium reduction of processed foods is still required and continuous monitoring of the reduction of sodium levels in processed foods is needed.

## 1. Introduction

The high amount of dietary salt, 9–12 g/day [1], consumed by populations in most countries in the world has been a health issue for decades. This is due to its well-established association with hypertension, which is a major determinant of cardiovascular diseases [2]. In response to the urgency of a reduction in dietary sodium, the World Health Organization (WHO) initiated a global movement. In 2013, all Member States agreed to the target of a 30% reduction in salt intake with the aim of reaching <5 g of salt per day by 2025 [3].

In Australia, the national government and other health authorities have developed and implemented strategies with the aim to reduce the amount of dietary salt. It has been estimated that processed foods contribute to 80% of dietary sodium in Australia [4] and reduction of sodium use in the food industry has been identified as the main approach for addressing this issue. The National Heart Foundation’s Tick Program that was implemented over 20 years ago has been successful over the years [5]. Many food companies have taken on the challenge of reducing the salt content of their products in order to meet the “Tick” criteria, which is dependent on the product category [5]. The Australian Division of World Action on Salt and Health (AWASH) and the Australian government’s Food and Health Dialogue (FHD) have both published sodium reduction targets. These organizations have been involved in acquiring major food companies to commit to reducing the amount of sodium used in their products until reformulation targets are met [6,7]. The sodium targets (as shown in Table 1) are 400 mg/100 g across bread products, 15% reduction for breakfast cereals exceeding 400 mg/100 g, 1090 mg/100 g for cured meats, 830 mg/100 g for luncheon meats, 10% reduction for wet savoury pies exceeding 400 mg/100 g and dry savoury pies exceeding 500 mg/100 g, 550–800 mg/100 g for potato chips, 950–1250 mg/100 g for extruded snack products, 850–1100 mg/100 g for salt and vinegar snack products, 850 mg/100 g for plain crackers, 1000 mg/100 g for flavoured crackers, 710 mg/100 g for cheddar cheese, and 1270 mg/100 g for other chilled processed cheese [7].

To ensure that the progress towards the sodium reduction targets is made, regular monitoring is essential. There has been a lack of analytical monitoring of sodium changes in processed foods throughout the years. Maples et al. [8] and Wills and Duvernet [9] conducted studies on the sodium content of Australian processed foods in 1980 and 1995, respectively. Both of these studies were conducted prior to the introduction of mandatory nutrition information panel labelling being published in the Commonwealth of Australia Gazette, No. P 30, Wednesday 20 December 2000, as part of Australia New Zealand Food Authority Amendment No. 53 to the Food Standards Code and came into effect from 20 December 2002 [10]. Wills and Duvernet [9] published a comparison of the analytical data from the two studies that presented approximately 10% sodium reduction over the 15 years interval. However, Wills and Duvernet [9] analysed composite samples comprised of all brands of similar food types rather than each product individually, rendering comparison difficult.

Thus, the main purpose of this follow-up study was to determine by chemical analysis the current sodium levels in the same or equivalent processed foods previously analysed. With the introduction of mandatory nutrition information panels [10], it was also considered appropriate to compare the analytical data obtained in the 2013 study with the sodium value declared on the food label.

## 2. Materials and Methods

The sodium content of 130 different processed foods available in the Australian supermarkets was determined in 2013. The food products were predominately selected based on the availability of those previously analysed by Maples et al. [8] and Wills and Duvernet [9]. The brand and product name of all processed foods were obtained and a market survey was conducted to determine the availability of the products. Products that were no longer obtainable were substituted with an equivalent product. An alternative product by the same manufacturer was selected if available, otherwise the most equivalent product was chosen. The majority of the products were branded products produced by various different manufacturers but some supermarket branded products were also included in the analysis. Two purchases were made of each food product from two different supermarkets in Sydney, ensuring that they were stamped with different batch numbers. The food products were analysed individually, but the results were separated into seven categories: processed meat products, convenience foods (pizza, meat pie, and sausage roll), cheese, bakery products, breakfast cereals, muesli, and snack foods.

Prior to analysis, equal quantities of the two samples purchased for each product were blended to obtain a composite sample with a mass of 100g or more. Sub-samples (0.5 g) of each composite sample were covered with 5 mL of 70% nitric acid and left overnight. They were then heated to 80 °C until the sample became clear in colour (1–3 h) and then cooled prior to adding 2 mL of 30% hydrogen peroxide. The samples were allowed to stand until effervescence ceased before heating for 30 min at 110 °C. They were then made to volume (30 mL) using MilliQ water and left overnight before transferring to 10 mL tubes for analysis by inductively coupled plasma-optical emission spectroscopy (ICP-OES) [11]. Three different blank solutions were also prepared to assist in the calibration of the ICP-OES instrument by negating the effects of background. This method of analysis was used to be consistent with the 1980 and 1995 studies [8,9]. To ensure that the food products were representative of the food as consumed, heating or cooking required by some products was conducted according to the manufacturer’s instructions before homogenization. The Standard Reference Material (SRM) 1548a Typical Diet developed by the National Institute of Standards and Technology (NIST) was used to validate the analytical method used for the determination of the sodium content of the food products [12]. 

Analysis of Variance (ANOVA) and *t*-tests were used for data collected in 2013 as they followed normal distribution for individual category of food products. Hence, mean and range values along with the median values are being reported. For 2013 overall data (Table 1), there were small and varying sample sizes for individual product categories (e.g., 3 salami products; 10 samples each in the pizza, breakfast cereals or sliced white bread categories), normality tests have little power to reject the null hypothesis (that “sample distribution is normal”). Therefore, only results with small sample sizes passed the normality test [13] and have undergone parametric analysis in this instance. 

For samples collected from 1980, 1995, and 2013 which were 96, 63, and 116 respectively (Table 2), which represent a large enough sample size (>30 or 40), the normality assumption was not followed [14]. According to the central limit theorem, in large sample sets (>30 or 40), the sampling distribution tends to be normal regardless of the shape of the data [15,16] and means of random samples from any distribution (in this case for Table 2, where mean values from different product categories have been provided) would themselves have a normal distribution [17]. Hence, for all these above reasons, parametric tests have been used in this research.

Independent sample *t*-tests were used to compare the differences in sodium content between 1980 and 2013 overall and by each product category. This data was analysed using the Minitab 17 Statistical Software [18]. The 1995 data could not be included in the statistical analysis as composite samples had been analysed. The 2013 flatbreads and natural muesli data were also excluded from the statistical analyses to ensure products from both data sets included in the analysis were comparable. 

## 3. Results

**Figure 1:**
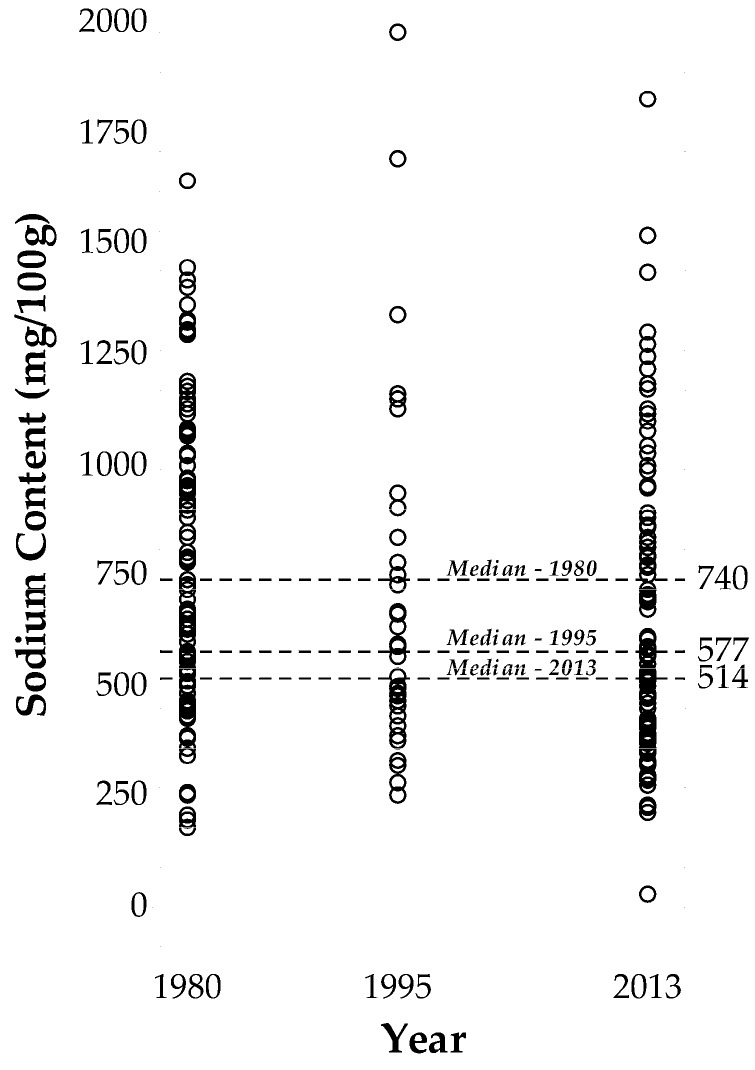
Comparison of the sodium contents of Australian processed foods between 1980 and 2013 where a circle represents each product.

The sodium contents of the Australian processed foods analysed in 2013 indicate that 62% of the products were compliant when compared to the set targets (Table 1). Overall, the sodium content of the processed foods studied decreased by 23%, 181 mg/100 g (*p <* 0.001, 95% CI, 90 to 272 mg/100 g), since 1980 with most of the reduction (12%, 83 mg/100 g) occurring between 1995 and 2013 (Table 2). Figure 1 indicates an increased density of products appearing toward the lower end of the sodium content scale in 2013 compared to previous years. Table 2 shows the changes in sodium content amongst the different food groups varied considerably with sodium levels of convenience foods (pizza, meat pie, and sausage roll) and cheese increasing by 4% and 50%, respectively.

### 3.1. Sodium Content of Processed Foods in 2013

In 2013, the average sodium content of Australian processed foods was 617 mg/100 g (Table 2). The most significant overall sodium reductions were in the convenience foods (pizza, meat pie, and sausage roll), 27% (*p* < 0.001, 95% CI, 94 to 291 mg/100 g) and breakfast cereals, 50% (*p* = 0.001, 95% CI, 218 to 593 mg/100 g) categories. The muesli category showed a 92% reduction, however it could not be tested for significance due to the low sample size. Conversely, only a slight decrease in the processed meats category, 3% (*p* = 0.655, 95% CI, −121 to 190) was seen, and this was not significant. The results also revealed an 8% (*p* = 0.781, 95% CI, −484 to 369 mg/100 g) sodium increase in the cheese category and this was also not significant. Furthermore, products in the sub-categories within the seven categories presented greater variations in sodium content (Table 1). Among the processed meats, sodium values ranged from 793 mg/100 g in canned luncheon meat up to 1297 mg/100 g in salami. Within the convenience foods group, sausage rolls (698 mg/100 g) contained the highest sodium level and meat pies (395 mg/100 g) contained the lowest. The sodium content of cheese varied greatly, ranging between 267 and 1828 mg/100g overall with processed cheeses—which are typically produced by blending one or more natural cheeses of different ages, emulsifying salts, water, other dairy, and non-dairy ingredients [20]—containing the most sodium. Of the bakery products, crackers had the highest average sodium content of 743 mg/100 g, with the other biscuit subcategories having an average between 338 and 447 mg/100 g. Despite being characterised as “sweet”, it is interesting to see that sweet biscuits have high sodium contents. The average sodium level in multigrain (403 mg/100 g), white (390 mg/100 g), and wholemeal (393 mg/100 g) bread was shown to be quite similar. However, the fruit bread analysed was considerably lower with a sodium content of 220 mg/100 g. In comparison to the other types of bread, white (472 mg/100 g) and wholemeal flatbreads (497 mg/100 g) contained the most sodium on average. The cheesecake subcategory had a mean sodium content that is one of the lowest in the bakery products group at 219 mg/100 g. The mean sodium content of breakfast cereals was 401 mg/100 g, which can be considered to be high when evaluated against muesli (16 mg/100 g). In the snack foods category, extruded snack foods had the highest average sodium content, 964 mg/100 g, compared to potato chips (720 mg/100 g) and potato straws (764 mg/100 g).

### 3.2. Changes in Mean Sodium Content between 1980 and 2013

The changes in the mean sodium content of processed foods between 1980 and 2013 are shown in Table 2. The average sodium content of processed meats in 2013 was lower than the values published in 1980 and 1995 by 3% (*p* = 0.655, 95% CI, −121 to 190) and 7%, respectively, though this was not statistically significant. For convenience foods (pizza, meat pies, and sausage rolls), when compared with the 1980 average sodium content, a 27% (*p* < 0.001, 95% CI, 94 to 291 mg/100 g) reduction is indicated, though there appeared to be a 4% sodium increase between 1995 (by data inspection only) and 2013. The mean sodium content of cheeses is shown to have increased by about 8% (*p =* 0.781, 95% CI, −484 to 369 mg/100 g) since 1980, apparently doubling since 1995, although this was not statistically significant. Continual reduction of sodium used in bakery products was evident, with an overall but not significant reduction of approximately 17% (*p* = 0.115, 95% CI, −22 to 192). Conversely, the sodium content of breakfast cereals had declined significantly by 50% (*p* = 0.001, 95% CI, 218 to 593 mg/100 g). The greatest reduction has occurred within the muesli category, with a 92% decrease. Although the muesli category consisted of a low sample size, the products analysed in 1980 were the same brands re-analysed in 1995 and 2013. Since 1980, the sodium content of snack foods had decreased by 21% (*p* = 0.017, 95% CI, 44 to 398 mg/100 g) though only a 2% reduction may have occurred since 1995 (using data inspection only). Overall, the mean sodium content of processed foods continued to decline over the years (Table 2).

### 3.3. Proportion of Processed Foods that Met Established Sodium Targets in 2013

The proportion of all Australian processed foods analysed in 2013 that met sodium targets established by the FHD and AWASH was 62% (Table 1). All of the products categorised under natural cheddar cheese, cheesecake, muesli, potato chips (salt and vinegar), potato straws, and extruded products complied in 2013 with the reformulation targets. However, none of the pizza (frozen/refrigerated), sausage roll, or sweet biscuit products met the set targets. The majority of the other categories had ≥50% of products meeting the reformulation targets.

### 3.4. Comparison of Analytical Sodium Content Data with Label Claims in 2013

The majority of the discrepancies between the actual analytical and label-declared sodium contents of processed foods were within the suggested acceptable range of ±20% [21], with 14% of products being outside the acceptable range (Table 3). The extent of the sodium content variation of both muesli products analysed when compared to the label-declared sodium values were unacceptable, with a 26% under-declaration for the toasted muesli and a 65% over-declaration for the natural muesli. When comparing the analysed and declared sodium content values of convenience foods, 24% of products were shown to have unacceptable label discrepancies. These products were from the pizza sub-category and the differences ranged from 25% less sodium to 26–43% more sodium than the label-declared amount. The breads and other bakery products category had 14% of products testing at unacceptable levels. The greatest discrepancies between analytical data and label claim were most prevalent in the flatbreads sub-category, which were shown to contain 28–47% less sodium than the value declared. The biscuits sub-category had the least amount of variation, ranging between 4% less than the amount declared to 8% more. All breakfast cereals were within ±20% of the sodium content declared on the label. The majority of products contained 1–18% less sodium than the content declared on the label, with a few products containing 2–10% more sodium than declared.

## 4. Discussion

The findings from this study indicate that Australian food companies are making efforts to progressively reduce the amount of sodium added to their processed food products. It is reassuring to observe that the sodium content of processed foods has continued to decline (12%) since the 1995 study. The overall reductions of 12% between 1995 and 2013, and 23% between 1980 and 2013 (*p* < 0.001, 95% CI, 90 to 272 mg/100 g) are positive results. The proportion of products meeting the reformulation targets set is also promising as 80 out of 130 products analysed were within the AWASH and FHD recommendations. Despite this positive result, it is apparent that further improvements are still required. This is achievable if greater emphasis were given to the importance of sodium reduction within the food industry. To maintain momentum, there must be continual monitoring of the progress towards sodium reduction targets using proper methods of reporting as this ensures that companies are fulfilling their pledges [22,23].

Sodium plays various important roles in many food products such as flavour, texture enhancement, and shelf life. This can make sodium reduction difficult when ensuring the acceptability is not affected. For thousands of years, sodium has been used in processed meats to assist in preservation by lowering the water activity and acts as a binding agent [24]. Similarly, salt is used in the production of cheese due to its ability to aid in moisture and microbial control in addition to being an emulsifier [20]. Given that the average sodium content of processed meats only decreased slightly and that there was a considerable increase of sodium in cheese suggests that limitations to the extent of sodium reduction may be responsible. The minimal reduction of sodium in meat products is quite concerning, considering this food group has been assessed as accounting for 21% of Australian’s salt intake [25]. However, the overall reduction is still reassuring seeing as Wills and Duvernet [9] previously reported a 30% increase in the sodium content of salami which was likely due to an industry response to the Garibaldi salami food poisoning incident in 1994. The most substantial reductions that have occurred since 1995 are in the bakery products (23%), breakfast cereals (42%), and muesli (90%) categories. This is a good outcome given that cereals and cereal products (grains, bread, pasta, biscuits, etc.) account for 32% of Australian’s salt intake and cereal based products and dishes (biscuits, muffins, pizza, etc.) accounted for 17% according to assessments by Webster et al. [25].

It is evident that there are issues and challenges that restrict the extent of sodium reduction achievable for different food groups. Consumer acceptance of sodium reduced products is important, especially when flavour is the major determining factor of food acceptance and consumption [26]. Studies have shown that sodium levels can be reduced by 30% to 50% without influencing the taste and consumer acceptability, where gradual reduction overtime is the key to minimising the noticeability of the change [27,28,29,30]. Excessive sodium reduction however, can lead to its replacement at the table where up to 20% can be added [26,31]. This is where consumer education and awareness can be extremely beneficial, where guidance is provided to assist consumers to change their habitual discretionary salt use. Following a national campaign in England, a study revealed that the number of consumers that added salt at the table had decreased by greater than 25% after five years [32]. Where sodium is important for its functional attributes, reduction may result in the requirement of additives to assist in achieving the same favourable characteristics of the product, which can be undesirable [33]. 

In light of the fact that the nutrition information displayed on the packaging of food products are typically generated theoretically using nutrition databases rather than from actual analysis, discrepancies between the values declared and the analysed contents were assessed. The sodium content of the foods analysed were predominantly within ±20%, which has been suggested to be an acceptable discrepancy range [21]. However, 14% of products were not within this acceptable limit. The issue may be simply due to the use of theoretical nutrition information on the labels. Manufacturers continuously reformulate products [34], therefore it is possible that with changes in the formulation of products, the nutrition labels have not been updated accordingly. Nutrition labels were introduced to assist consumers with their food choices by allowing them to know what they are consuming. It is important that industry declares nutrient content accurately to ensure that consumers can trust the nutrient values declared on labels [35]. 

## 5. Conclusions

In conclusion, the analytical data from this study indicates a declining trend in the sodium levels of Australian processed foods. The continual reduction of sodium used during the manufacturing of food products is highlighted, though further reduction is still necessary. Whilst the reduction of salt can reduce the sodium content of a product, it is important to also consider other components in processed foods that contribute to the overall sodium content. Technical limitations may be considered a challenge, however research into new technologies and solutions to the complications that may be faced can assist in developing innovations. The variations between the sodium content of products within categories such as processed meats (793–1297 mg/100 g) and cheese (267–1828 mg/100 g) suggest that reformulation to reduce the sodium content is possible. Gradual reduction of the sodium levels by the food industry will assist in adapting the preference of consumers [29]. The engagement of more food companies in the FHD and AWASH reformulation programs is essential for more consistent reductions. Ongoing salt reduction programs have been most effective in other countries such as the United Kingdom, the United States, Canada, and Finland where the government has been involved [36,37,38]. Regulation where non-compliance results in some form of penalty is believed to be a strong driver for industry reformulation by public health experts [22,39]. The implementation of mandatory reformulation targets to the entire food industry with the support of the Australian government would be highly beneficial. This would help support continual improvements and ensure that Australia is contributing to the global 30% reduction target set by the WHO. It is also imperative that nutrition information panels are updated when formulation changes are implemented so that consumers are provided with the correct information. However, given that the Australian regulations allow for the nutrition information panels to be theoretically calculated, the use of the sodium content on labels may not be entirely reliable. To track the progress of the food industry accurately and to better monitor the sodium reduction of processed foods, analytical data should be obtained on a regular basis. Conducting future studies to obtain analytical data on the sodium content of the same or similar processed foods included in this study will allow for continuous monitoring of the changes in sodium content in relation to reformulation targets and food labelling. Alternatively, focusing on processed food categories that contribute the most to daily salt consumption—such as bread, bread rolls, processed meat, and cereal products [40]—could allow for more frequent random analysis and comparisons against nutritional information panels to be feasible.

## Figures and Tables

**Table 1:** Sodium content of Australian processed foods in 2013.

	Sodium Content (mg/100 g)				
	No. of Products	Mean	Range	Target	% of Products Meeting the Target
Processed meats					
Salami	3	1297	1181–1435	1400 ^†^	67%
Ham	4	1131	864–1520	1090 ^‡^	50%
Corned beef, canned	1	813		830 ^‡^	38%
Luncheon meat, canned	1	793			
Luncheon knobs	5	943	788–1218		
Liverwurst	1	859			
Frankfurts	5	1056	862–1299	1150 ^†^	80%
Convenience foods					
Pizza	10	557	412–834		
Frozen/refrigerated	6	540	416–692	390 ^†^	0%
Take-away	4	582	412–834	530 ^†^	25%
Meat pie	6	395	354–444	400 ^‡^	50%
Sausage roll	1	698		450 ^†^	0%
Cheese					
Natural cheddar cheese	2	680	670–689	710 ^‡^	100%
Other natural cheese	7	597	276–1075	1270 ^‡^	89%
Processed cheese	2	1535	1242–1828		
Breads and other bakery products					
Crackers	5	743	572–1024	850 ^‡^	88%
Crispbread	3	339	227–513		
Sweet biscuit, plain	4	447	285–523	270 ^†^	0%
Sweet biscuit, cream	2	338	291–384	170 ^†^	0%
Bread, multigrain, sliced	7	403	358–474	400 ^‡^	65%
Bread, white, sliced	10	390	346–511		
Bread, wholemeal, sliced	9	393	321–519		
Fruit bread, sliced	1	220			
Flatbread, white	7	472	151–902		
Flatbread, wholemeal	6	497	163–882		
Cheesecake	2	219	214–223	240 ^†^	100%
Breakfast cereals					
	10	401	288–563	400 ^‡^	60%
Muesli					
Natural	1	8			
			400 ^‡^		100%
Toasted	1	24			
		Snack foods			
Potato chips	8	720	483–996	800 ^‡^	75%
Salt and vinegar	2	874	751–996	1100 ^‡^	100%
Potato straws	1	764		800 ^‡^	100%
Extruded products	5	964	674–1169	1250 ^‡^	100%
				Overall	62% (80/130)

^†^ The Australian Division of World Action on Salt and Health target [19]; ^‡^ The Food and Health Dialogue target [7].

**Table 2:** Changes in sodium content of Australian processed foods between 1980 and 2013.

	2013	1995 [9]	1980 [8]	Sodium Content Change (mg/100 g)			
				1995–2013		1980–2013	
				mg	%	mg	%
Processed meats							
No. of products	20	15	24				
Mean sodium content (mg/100 g)	1043	1120	1078	−77	−7%	−35	−3%
Convenience foods (pizza, meat pie, and sausage roll)							
No. of products	17	8	16				
Mean sodium content (mg/100 g)	508	490	700	+18	+4%	−92	−27%
Cheese							
No. of products	11	11	8				
Mean sodium content (mg/100 g)	782	520	725	+262	+50%	+57	+8%
Breads and other bakery products							
No. of products	43	17	23				
Mean sodium content (mg/100 g)	421	550	506	−129	−23%	−85	−17%
Breakfast cereals							
No. of products	10	7	10				
Mean sodium content (mg/100 g)	400	684	806	−284	−42%	−406	−50%
Muesli (toasted)							
No. of products	1	1	2				
Mean sodium content (mg/100 g)	24	250	317	−226	−90%	−293	−92%
Snack foods							
No. of products	14	4	13				
Mean sodium content (mg/100 g)	810	830	1032	−20	−2%	−222	−21%
Overall							
No. of products	116	63	96				
Mean sodium content (mg/100 g)	617	700	798	−83	−12%	−181	−23%

**Table 3:** Food label discrepancies between the analytical and label-declared sodium contents of processed foods analysed in 2013.

Food Category	Unacceptable ^
Processed meats	10% (*n =* 2/10)
Convenience foods	24% (*n =* 4/17)
Cheese	9% (*n =* 1/11)
Breads and other bakery products	14% (*n =* 8/56)
Breakfast cereals	0% (*n =* 0/10)
Muesli	100% (*n =* 2/2)
Snack foods	7% (*n =* 1/14)
Overall	14% (*n =* 18/130)

^ Unacceptable when exceeding the suggested acceptable discrepancy range of ±20% [21].
